# Complications in blepharoplasty: how to avoid and manage them

**DOI:** 10.1590/S1808-86942011000300009

**Published:** 2015-10-19

**Authors:** Tomas Gomes Patrocinio, Bruno Alvarenga Silva Loredo, Carlos Eduardo Arnez Arevalo, Lucas Gomes Patrocinio, José Antonio Patrocinio

**Affiliations:** 1 Otorhinolaryngologist; Head of the Ocular Plastic Surgery Division - Otorhinolaryngology Department - Medical School of the Federal University of Uberlândia; 2 Resident physician - Otorhinolaryngology Department - Medical School of the Federal University of Uberlândia; 3 Resident physician - Otorhinolaryngology Department - Medical School of the Federal University of Uberlândia; 4 PhD - Medical School of São José do Rio Preto, Head of the Maxillo-Facial Surgery Division - Otorhinolaryngology Department - Medical School of the Federal University of Uberlândia; 5 Full Professor - Otorhinolaryngology Department - Medical School of the Federal University of Uberlândia; Divisão de Plástica Ocular, Serviço de Otorrinolaringologia, Universidade Federal de Uberlândia, Uberlândia, Minas Gerais, Brasil

**Keywords:** blepharoplasty, postoperative complications, eyelid diseases, eyelids

## Abstract

Complications in blepharoplasty are uncommon and, when they occur, they are usually mild and transient, such as hematomas and chemosis. However, sometimes they can be severe, such as blindness, or they might require surgical correction, such as ectropion.

**Objective:**

To evaluate the results and complications of transcutaneous blepharoplasty performed in the same procedure, discussing how to treat and how to avoid them.

**Methods:**

A retrospective study of 200 medical records of consecutive patients that underwent transcutaneous blepharoplasty from January 2007 to January 2009. The variables analyzed were age, gender, complications, clinical outcome, patient satisfaction, and photographic documentation.

**Results:**

The incidence of complications was 9.5% (19 patients). The complications were 1 hematoma, 12 cases of chemosis and 13 patients who underwent canthoplasty, 6 patients with malposition of the lower eyelid (5 retractions and 1 ectropion). Medical treatment was performed in 12 patients and revision surgery in 7 cases of all the patients who had complications.

**Conclusions:**

We demonstrated that blepharoplasty is a procedure with a high satisfaction and a low complication rate, and it is an excellent surgical procedure, when properly indicated.

## INTRODUCTION

Blepharoplasty is among the most performed cosmetic surgeries in the world today, and its main goal is to promote rejuvenation, functional and cosmetic enhancement of the periorbital region^1^.

Complications are uncommon and when they happen they are usually mild and transitional, such as hematomas and chemosis. Nonetheless, sometimes they can be definitive, such as blindness, or they require new surgical approaches for correction, such as ectropion and eyelid ptosis^2^.

Complication prevention or even their forecast starts with a careful preoperative assessment. It must include a detailed clinical history (comorbidities, use of medication, ophthalmic past, personal and family members), and careful physical exam. The assessment of the patient's psychological aspects and the patient's expectations regarding surgery are also very important. The surgical technique to be used is based on the anatomical changes found and the patient's complaints, always taking into account patient expectation and the real surgical possibilities for cosmetic improvement^3^.

The treatment of complications include from a clinical approach all the way to new surgical interventions, which must be properly employed by face surgeons properly trained to do a blepharoplasty.

The goal of the present paper is to assess the results and complications of transcutaneous blepharoplasties done by the Ocular Plastic Surgery service from the Otorhinolaryngology Department from a University Hospital, from January of 2007 through January of 2009, showing how to treat them and, especially, how to avoid them.

## MATERIALS AND METHODS

### Series

We reviewed the charts from 200 consecutive patients submitted to transcutaneous blepharoplasty surgery between January of 2007 and January of 2009 in an otorhinolaryngology department in a university hospital. The variables analyzed were: patient age and gender, the surgical procedure (duration, anesthesia and surgical technique), complications (hematoma, chemosis, lower eyelid mal-positioning, lagophthalmos, epicanthus, eyelid ptosis, eyebrow ptosis, senile orbit, diplopia) and periodic clinical evolution carried out in the outpatient ward during one year after the procedure, emphasizing the degree of patient satisfaction and pre and post-operative pictures.

All the surgeries were carried out under supervision of the same surgeon. The procedures were done under local anesthesia with local injection of lidocaine with epinephrine at 1:100.000 concentration in the place of incision and for a regional block. After heart and peripheral oximetry were monitored, the patient was sedated with the slow intravenous injection of 2ml of fentanyl chlorhydrate and 3 mg of midazolam.

### Surgical Technique

The upper blepharoplasty technique used was:

• The eyelid incision was marked with the patient seating down and looking straight ahead to a fixed point, highlighting the excess tissue to be resected;
• Upper eyelid incision with the 15 blade scalpel in the highlighted place;
• Removal of a strip of orbicular muscle from the eye, its pre-septal portion and the fat pouches are dissected;
• The excess fat in the pouches are excised and cauterized with the bipolar cautery;
• Intradermal continuous skin suture with 6-0 nylon wire.
  The lower blepharoplasty surgical technique was:
• Creation of the myocutaneous flap by a subciliary incision;
• Careful removal of the excess fat, muscle and skin;
• Canthopexy or canthoplasty, depending on the degree of lower eyelid laxity and that of the canthal tendon;
• Orbit oculoplastics;
• Skin suture with simple 6-0 nylon suturing wire.

## RESULTS

Of the 200 patients included in the study, 81% (162) were females and 19% (38) were males, with mean age of 44.5 years (35 to 60).

**Graph 1:**
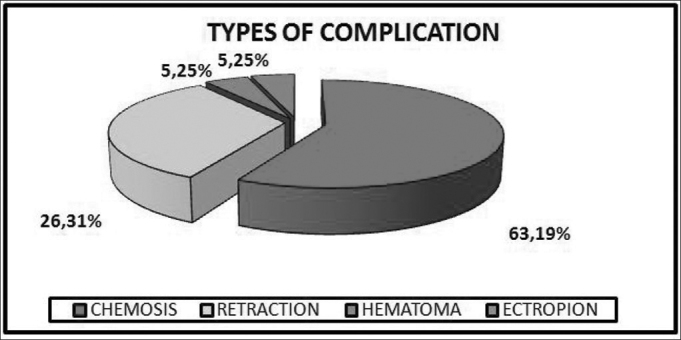
Graph showing the distribution of the main complications happened to patients submitted to blepharoplasty.

The incidence of complications was 9.5% (19 patients). The complications found were: 1 patient (0.5%) with hematoma; 12 cases (6%) of chemosis, and this happened to 61.5% of the 13 patients submitted to canthoplasty; 6 patients (3%) with lower eyelid malpositioning and from these, 83% corresponded to retraction (2.5% from the total, 5 patients) and 17% to ectropion (0.5% of the total, 1 patient) (Graph 1).

All the patients who had lower eyelid malpositioning were not submitted to lateral canthal anchoring in the primary surgery. We did not find some of the complications reported in the literature, such as: eyelid closure changes, lagophthalmos, epicanthus, eyelid ptosis, eyebrow drop, senile orbit and diplopia.

As far as the management of complications is concerned, a clinical treatment was carried out with outpatient follow up in 12 patients (63%) and revision surgery in 7 patients (37%) of all the patients with complications. The revision surgeries happened because of a hematoma in one patient and 6 patients were submitted to a second look because of the malpositioning of the lower eyelid border.

Of the 200 patients submitted to the surgical procedure, 6 had cosmetic complaints (3%) after surgery because of the lower eyelid mal-positioning and consequent apparent sclera. They were submitted to revision surgery and were pleased with the outcomes.

## DISCUSSION

Blepharoplasty is a surgery with rare complications; nonetheless, when they happen they may cause important functional and/or cosmetic damage. Nonetheless, besides being rare, most of the times these complications are simple to manage when treated by an experienced surgeon with anatomical knowledge of the eyelid and orbit regions^4^.

Among possible complications we stress hematoma, chemosis, lower eyelid mal-positioning, eye closure deficit, epicanthus, lagophthalmos, eyelid ptosis, eyebrow ptosis, senile orbit and diplopia^1^, ^5^.

### Hematoma

Hematoma is a complication that, depending on its magnitude and evolution, may cause amaurosis. Its prevention starts with preoperative care, a detailed clinical history involving coagulopathies, comorbidities which may predispose to bleeding and the use of anticoagulant drugs^6^, ^7^.

They may be classified as:

(1) Pre-septal: confined to the eyelid alone, not causing any risk to vision and resolving with ice packs.
(2) Post-septal: it happens because of intraorbital hemorrhage, causing a pressure increase in the orbit and compromising the optical nerve.

Demere and Wood reported 40 cases of blindness caused by retrobulbar hematoma in a series of 98,514 blepharoplasties (1 for each 2,500 procedures = 0.04%)^4^.

Drugs which predispose to hemorrhage are antiplatelet adhesion drugs and anti-inflammatory agents - which must be suspended at least 10 days before the procedure. Chronic disorders such as systemic arterial hypertension, must be controlled before surgery.

During surgery, hemorrhage control must be rigorous, with the cauterization of the fat pouches resected in order to avoid retrobulbar hematoma, as well as the cauterization of bleeding points in the orbicular muscle and adjacent tissues. There are controversies about the type of coagulation that must be used during surgery^1^, ^8^. Most of the authors prefer to use the bipolar cautery to avoid the risk of damaging posterior eye structures.

In cases of small hematomas, without visual or pupillary changes, one must be very conservative, using systemic steroids and strict follow up.

In cases of pupillary and visual changes, with the tense orbit, intervention must be immediate, with surgical exploration for hemostatic purposes. Depending on the hematoma severity, one must do a canthotomy, cantholysis or orbit decompression.

Post-operative strong pain is extremely rare, and when it happens one must consider the possibility of a hematoma formation. Routinely, one must avoid compressive dressing, and ice packs are used.

In our investigation, there was one case of hematoma (0.5% of all the patients), in which the patient had a fast evolution, with a large bulging which drew our attention for a surgical approach before there were pupillary or visual changes. This case shows the importance of also assessing the time it took the hematoma to form, its magnitude and evolution. Results are satisfactory if treatment is instituted early on.

### Chemosis

**Figure 1:**
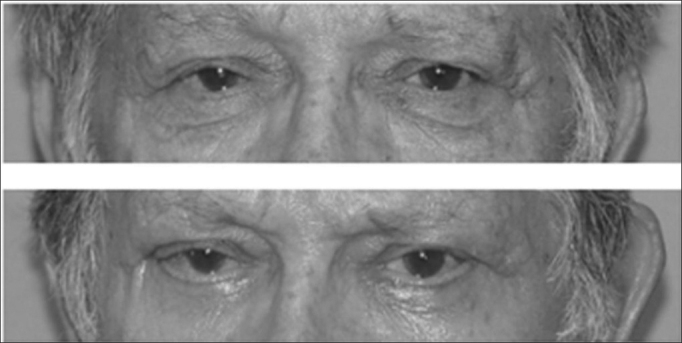
Patient with bilateral chemosis after blepharoplasty with canthoplasty.

Chemosis is an infiltrative edema of the eye conjunctive. It results from liquid collection arising from inflammatory reaction, lymphatic drainage obstruction and deficient eye closure, originating a bulged and reddish border around the cornea. In our series, chemosis was the most common complication (6% of all the patients) (Figure 1). It happened more frequently after surgery of the lower eyelid, especially when canthoplasty was carried out, because of the intense handling of the conjunctiva. Spontaneous resolution happened within 3 to 4 weeks, and a vasoconstrictor and steroid eye drops may be used in order to speed up the process^1^, ^5^.

### Lower eyelid mal-positioning

The ideal position of the lower eyelid is at 1mm or above the lower limbus. The lower eyelid malpositioning was the second most common complication found in our patients. The main causes of lower eyelid malpositioning are excessive skin resection (anterior lamella); inadvertent scarring of the orbital septum (middle lamella)^9^; and the failure in cantus anchoring^10^, ^11^, ^12^, ^13^, ^14^.

In order to prevent lower eyelid malpositioning, it is important to be careful in removing the excess skin on the lower eyelid; to suture the orbital septum, reducing the possibilities of an inadvertent scarring; and to anchor the lateral cantus, either with a canthopexy or cantoplasty^1^, ^5^, ^15^, ^16^

In order to correct the lower eyelid malpositioning, one must anchor the eyelid cantus associated with the orbit oculoplasty with a suspension of the myocutaneous flap, free skin graft or upper eyelid flap in some cases, and even use a posterior lamella expander.^9^, ^16^

Some anatomical characteristics are true traps, which increase the risk of malpositioning and the surgeon should treat them more carefully. They are: elderly patients with laxity and deep eye sockets; and patients with bulging eyes and negative vector (anterior portion of the eye bulb anterior to the lower eyelid and to the malar promontory)^16^, ^17^, ^18^.

**Figure 2:**
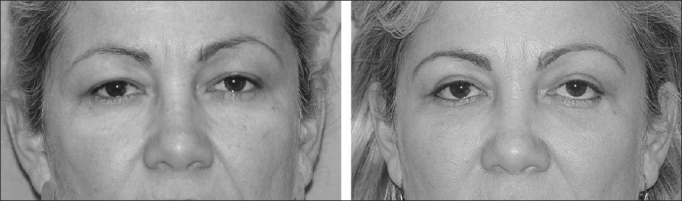
Patient with left lower eyelid malpositioning after blepharoplasty caused by retraction.

**Figure 3:**
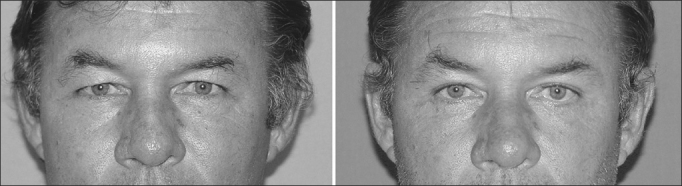
Patient with left lower eyelid malpositioning after blepharoplasty caused by retraction.

**Figure 4:**
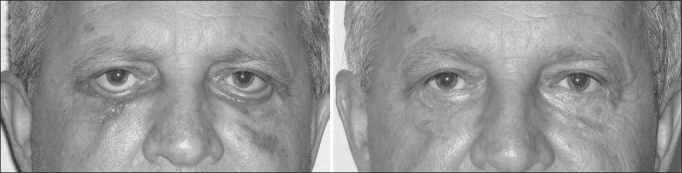
Patient with lower eyelid malpositioning after blepharoplasty caused by ectropion. Below: postoperative picture after correction by means of canthoplasty with the tarsal strip and skin grafting.

In the present study, the malpositioning cases happened because of retraction and ectropion (Figures 2, 3 and 4). In such cases we did not do any type of lateral canthal anchoring in the primary surgery. The retraction correction was carried out by means of the canthoplasty using the McCord technique (full thickness), and the ectropion was corrected using a tarsal strip associated to a skin graft, achieving good post-operative results^11^, ^12^.

### Lagophthalmos

The eyelid slit had 10mm of distance between the upper eyelid and the lower one, and 30mm of distance between the medial and the lateral canthi. Lagophthalmos is an alteration in which the eye cannot be fully closed, usually because of an excessive retraction of the upper eyelid tissue, orbicular muscle paralysis or lower eyelid retraction.

An inadvertent lesion of the upper eyelid elevator muscle aponeurosis may cause eyelid ptosis, also changing inter-eyelid distance. Lateral canthoplasty may reduce the distance between the medial and lateral canthi.

It is the most common cause of dry eye after blepharoplasty and in some cases it is necessary to operate the patient in order to correct the situation, doing a tarsorrhaphy, skin grafts and middle third suspension. Dry eye may be treated with massage and artificial tear drops, and most of the times it resolves spontaneously. We did not have this complication in our sample^1^, ^5^, ^19^, ^20^.

### Eyelid ptosis

Eyelid ptosis is a condition in which the upper eyelid falls below its normal level and the upper eyelid elevator muscle is not able to raise it. It is classified in two major groups: congenital or acquired^21^, ^22^. Preoperative assessment is very important, one should always look for ptosis, because it may become even more apparent after blepharoplasty is done, frustrating patient's expectations both in the cosmetic and in the functional aspects. The ptosis mechanism can be: myogenic, neurogenic, mechanical or aponeurotic.

The main cause of postoperative ptosis is eyelid edema, which causes a mechanical restriction to the eyelid elevator muscle action, with spontaneous resolution in most of the cases. Hematomas may also cause restrictions to the eyelid elevator muscle movement, and when deep and persistent it may cause fibrosis, making the situation last longer or even become permanent, although most of the times it resolves spontaneously. Ptosis may happen if the orbital septum is inadvertently joined to the elevator muscle during blepharoplasty suture, as well as forming adherences between the septum and the eyelid elevator, in cases of careless removal of the post-septal fat pouches. Damage to the eyelid elevator aponeurosis with the cautery may also cause ptosis.

Most of the ptoses cases resolve spontaneously within 3 months. When it does not happen, the patient must be submitted to surgery. The correction of post-surgery eyelid ptosis, usually the result of injury to the eyelid elevator muscle aponeurosis, involves the exploration of the upper eyelid and aponeurosis repair^3^, ^22^, ^23^, ^24^.

### Eyebrow ptosis

Eyebrow ptosis must be assessed in the preoperative visit. The patient must be educated as to the limitations of blepharoplasty in cosmetic improvement and possible complications, such as a reduction in the distance between the eyelid border and the eyebrow. Surgical options to reposition the eyelid must be offered to the patient by means of an endoscopic or transpalpebral surgery.

When the surgeon disregards this complication, the patient will be dissatisfied with the blepharoplasty's cosmetic results, and the eyebrow ptosis can be misjudged by the patient as being a complication arising from inadequate surgical technique^25^, ^26^, ^27^.

### Senile orbit

As time goes by, there is a loss of support and also a reduction in facial soft tissue volume. This is extremely important because as one performs blepharoplasty, the excessive removal of fat pouches can increase upper and lower eyelid grooves, giving the patient the appearance of aging. This situation may not be perceived in the immediate post-op, becoming more evident in the long run, when the patient develops the so called “senile orbit”. Even in a younger patient, one must avoid the excessive removal of fat pouches, because the loss of soft tissue is part of natural aging and the excess fat removed will be missed in the future. The correction of such complication can be done by the surgical technique described by Maniglia et al^28^.

### Diplopia

Diplopia, or double vision is a rare alteration which can happen in cases involving the muscles responsible for eye movement, or scars around the eyeball. The most vulnerable muscle is the inferior oblique. It may be transitional or permanent, requiring surgical correction when it does not evolve properly^1^, ^2^.

### Unhappy patient

Patient satisfaction with the blepharoplasty result depends on the following points: 1) detailed preoperative assessment; 2) contraindication of the cases which patient expectation is unreal; 3) indication of the most adequate surgical technique, including possible associations such as eyebrow elevation, ptosis correction, canthoplasty, and others; 4) carefully performed surgical technique, especially in hemostasis and the resection of skin and fat; 5) frequent postoperative follow up^3^, ^18^.

In the present study, there was a high patient satisfaction rate, especially because the surgeons followed the points listed above. The complications found are within the regular rates published in the world literature and could be corrected by clinical or surgical management.

## CONCLUSION

Blepharoplasty is a surgery not only with a cosmetic component, making the person's look younger and less tense, but it also has a functional component, providing the patient with a better visual field without the need to stress eyelid muscles. In order to achieve a good result, it is necessary to do a careful pre-operative evaluation, educating the patient as to the benefits and limitations of the procedure and also to customize the approach according to the characteristics of each patient. When surgery is carefully done, it entails few complications, thus guaranteeing its success.

The present study showed that blepharoplasty is a procedure with a high satisfaction rate and low number of complications, yielding excellent results when properly indicated.
